# Boosting Multilabel Semantic Segmentation for Somata and Vessels in Mouse Brain

**DOI:** 10.3389/fnins.2021.610122

**Published:** 2021-04-12

**Authors:** Xinglong Wu, Yuhang Tao, Guangzhi He, Dun Liu, Meiling Fan, Shuo Yang, Hui Gong, Rong Xiao, Shangbin Chen, Jin Huang

**Affiliations:** ^1^School of Computer Science & Engineering, Wuhan Institute of Technology, Wuhan, China; ^2^Engineering Research Center of Hubei Province for Clothing Information, School of Mathematics and Computer Science, Wuhan Textile University, Wuhan, China; ^3^Britton Chance Center for Biomedical Photonics, Wuhan National Laboratory for Optoelectronics-Huazhong University of Science and Technology, Wuhan, China

**Keywords:** artificial intelligence, deep learning, multilabel segmentation, boosting method, 3D reconstruction, convolutional neural network

## Abstract

Deep convolutional neural networks (DCNNs) are widely utilized for the semantic segmentation of dense nerve tissues from light and electron microscopy (EM) image data; the goal of this technique is to achieve efficient and accurate three-dimensional reconstruction of the vasculature and neural networks in the brain. The success of these tasks heavily depends on the amount, and especially the quality, of the human-annotated labels fed into DCNNs. However, it is often difficult to acquire the gold standard of human-annotated labels for dense nerve tissues; human annotations inevitably contain discrepancies or even errors, which substantially impact the performance of DCNNs. Thus, a novel boosting framework consisting of a DCNN for multilabel semantic segmentation with a customized Dice-logarithmic loss function, a fusion module combining the annotated labels and the corresponding predictions from the DCNN, and a boosting algorithm to sequentially update the sample weights during network training iterations was proposed to systematically improve the quality of the annotated labels; this framework eventually resulted in improved segmentation task performance. The microoptical sectioning tomography (MOST) dataset was then employed to assess the effectiveness of the proposed framework. The result indicated that the framework, even trained with a dataset including some poor-quality human-annotated labels, achieved state-of-the-art performance in the segmentation of somata and vessels in the mouse brain. Thus, the proposed technique of artificial intelligence could advance neuroscience research.

## Introduction

3D digital reconstruction of the mouse brain from 2D image stacks is well known for its complexity and time-consuming nature due to the extremely high density of the vasculature and neural networks in brains (Motta et al., 2019). Some automated neuron localization and tracking methods have been developed to accelerate the reconstruction speed with substantial success (Quan et al., 2013; Peng et al., 2017), whereas the complicated morphology and the dense distribution currently make a fully automatic and systematic framework of accurate 3D reconstruction pipelines still out of reach.

Image analysis algorithms play a decisive role in pipelines, among which deep-learning-based methods [mainly via deep convolutional neural networks (DCNNs)] along with their substantial advances in recent years have demonstrated significant success with robust evidence in applications such as image classification, object detection and semantic segmentation (Lecun et al., 2015; Long et al., 2015; Falk et al., 2019; Moen et al., 2019). These DCNN-based methods were integrated into processes that generate connectomics with the supervision of human-annotated labels, indicating a more efficient and more accurate human-machine interactive method to produce larger reconstructed volumes of mouse brains in less time (Todorov et al., 2020). Consequently, the correctness and completeness of human-annotated labels for dense nerve tissues, i.e., neurons, somata and vessels, in 2D images acquired by either X-ray microscopy (XRM), light microscopy or EM are more important in these deep-learning-based methods than ever before because these labels are utilized to guide essentially both the learning stage and the performance assessment stage of these methods (Zeng et al., 2017; Haberl et al., 2018; Li T. et al., 2019).

However, the gold standard of human-annotated labels for dense nerve tissue is often difficult, if not impossible, to acquire and accumulate since domain knowledge, experience and time are all required for human experts to annotate nerve tissue correctly and completely (Giovanna et al., 2018). For instance, our previous experience indicates that it typically takes about 10 working hours for a trained undergraduate student to finish labeling a single neuronal wire with a length of 6–8 cm in a microoptical sectioning tomography (MOST; Wu et al., 2014) image stack with a mean accuracy of 0.90–0.95. Labeling vasculature structures in the brain requires similar efforts. It is thus understandable that the gold standard would never be sufficient to satisfy the pressing needs of the current deep-learning-based methods, which are often trained with hundreds or thousands of annotated image data. Even so, the resulting reconstructions of dense nerve tissue are still error-prone, affecting its scientific practicality (Motta et al., 2019). Most biomedical researchers who are ready to use data-hungry DCNNs for segmentation tasks thus encounter a realistic challenge due to the availability of a relatively large set of poor-quality or questionable annotated data and only a small set of high-quality data or gold standard data. To address this problem, various strategies have been proposed; for instance, semisupervised or weekly supervised learning is proposed to utilize fewer labels and achieve better results (Fang et al., 2019; Vorontsov et al., 2019; Zhou et al., 2019). In this work, an alternative approach, inspired by the Adaboost method (Freund and Schapire, 1997), is proposed to gradually adjust the poor-quality training data supervised by a well-performing DCNN, which was trained sequentially to pay more attention to those hard-to-learn instances.

Notably, previous DCNNs in biomedical image segmentation have mainly focused on the segmentation of a single object (Kong et al., 2019; Moen et al., 2019; Todorov et al., 2020; Wu et al., 2020). Consequently, if multiple objects existing in the same image need to be segmented, previous DCNNs would spend considerable time and require hardware usage to make training and prediction efforts. Because both somata and vessel structures occur at the same time in MOST image stacks, a multilabel semantic segmentation network was thus proposed in this study for its characteristics of training once and obtaining the segmentation result for multiple objects simultaneously.

**FIGURE 1:**
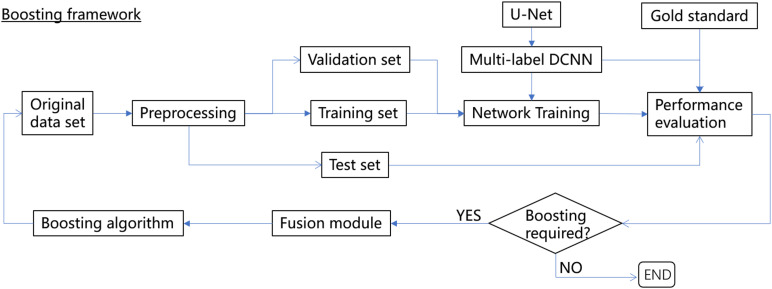
The boosting framework for multilabel semantic segmentation.

In this paper, we proposed a novel boosting framework (Figure 1) consisting of a multilabel DCNN based on U-Net (Ronneberger et al., 2015; Falk et al., 2019) with a customized Dice-logarithmic loss function, a fusion module combining the original human-annotated labels and the corresponding predictions from the DCNN, and a boosting algorithm to sequentially update the sample weights during network training iterations. The framework was then evaluated with the MOST dataset to achieve efficient and accurate segmentation of somata and vessel structures in the mouse brain. Considering that minor errors occur in human annotations, the proposed framework improved the network performance by about 3–10% for both somata and vessels with less prediction time. The main contributions of this work are summarized as follows:

• We developed a boosting framework to systematically improve the quality of human-annotated labels for deep-learning-based segmentation tasks.
• We formulated a customized Dice-logarithmic loss function for a multilabel segmentation network to mitigate the effects of ill-balanced classes in the training dataset without the introduction of extra hyperparameters.
• We performed experiments on MOST image stacks and demonstrated the advantageous performance in the segmentation of both somata and vessels compared to other methods.

Our source codes, the trained network weights, and a validation dataset are publicly available to better assist the development of a three-dimensional reconstruction of the mouse brain in the biological community.

## Related Work

### Biomedical Image Segmentation

Biomedical image segmentation has become essential in recent years due to the growing demand in life sciences and medicine, e.g., on the three-dimensional reconstruction of vasculature and neural networks in brains using microoptical data (Wu et al., 2014). Semantic segmentation has been applied to various scenarios. Arteries, veins, and capillaries have been reconstructed in the mouse brain (Xiong et al., 2017), as well as the retina in human eyes (Hu et al., 2018). In addition, the neural system is a composite of features, and different types of components should be segmented separately (Gong et al., 2016). Furthermore, brain tumors containing various tissues should be identified for accurate medical diagnosis (Kao et al., 2020).

However, most of the existing deep-learning-based segmentation techniques were developed to be single-label networks, i.e., to identify a single type of object in the images from the network output, including U-Net (Ronneberger et al., 2015; Falk et al., 2019), flood-filling networks (FFNs; Januszewski et al., 2018), DeepEM3D (Zeng et al., 2017), and CDeep3M (Haberl et al., 2018), which have achieved significant progress for segmentation tasks in different types of datasets, including light, X-ray, and electron microscopy (EM). These networks have achieved various degrees of success in the segmentation of dense nerve tissues. For instance, whole-brain mouse vasculature stained by two different dyes (i.e., wheat germ agglutinin and Evans blue) was reconstructed in four hours at human-level accuracy (about 0.94; Todorov et al., 2020). Combining the DCNN with the multitask learning method, an F1 score of 0.92 in somata segmentation was reported on a Nissl-stained dataset captured using the MOST system (Hu et al., 2021). A Docker-powered DCNN was employed for the segmentation of somata and vessels in MOST image stacks and achieved high accuracy on both tissues with F1 scores of 0.96 (Wu et al., 2020). Multilabel segmentation techniques can be developed based on existing single-label networks with multiple output branches but warrant further investigation (Hu et al., 2018), for instance, to properly accelerate the convergence of network training and consistently obtain a correct segmentation result from the designed output branch.

### Abnormal Annotation

Human annotated labels for natural images are far from perfect, and thus, several deep-learning-based methods have been developed to address these abnormal annotation issues. First, the coarse annotation issue has been partially resolved with a weakly supervised learning technique (Dietterich et al., 1997), which has been widely used for the segmentation of natural images (Oquab et al., 2015; Durand et al., 2017) and medical images (Hwang and Kim, 2016). However, the performance of weakly supervised learning for segmentation is known to be a challenge since the application of coarse annotations to networks of pixelwise predictions is laborious. Second, in the medical research field, clinical experts have often focused on specific anatomical structures and thus have produced partial or missing annotations (Petit et al., 2018). The issue could be largely leveraged by using a curriculum strategy (Bengio et al., 2009). Finally, noisy annotations are a typical challenge in machine learning (Natarajan et al., 2013), particularly in image classification and segmentation (Frenay and Verleysen, 2014; Algan and Ulusoy, 2019). Some noise-tolerant versions of CNNs have been developed (Lu et al., 2017; Li J. et al., 2019) and have achieved various degrees of success in public datasets such as Pascal VOC (Everingham et al., 2010) and CIFAR-10 (Krizhevsky and Hinton, 2009). However, to the best of our knowledge, the efficiency and accuracy reported in these studies are probably not adequate for the purpose of digital reconstruction of the brain, considering that human annotations for vasculature and neural structures in the mouse brain are even more laborious and error-prone. It is thus understandable that for segmentation tasks for somata and vessel structures in the brain with abnormal annotations, new techniques are still expected.

### Boosting-Related Methods in Image Segmentation

The acquisition of a large number of human annotations for biomedical images is always difficult and sometimes impractical, and thus, various strategies and techniques have been explored either to boost the size of the dataset or to boost network performance with the help of prior knowledge. For instance, to increase both the size and the diversity of the training dataset, human annotations from other domains, e.g., BBox (bounding box) and ROI (region of interest), are utilized in a weakly supervised mechanism (Dai et al., 2015; Gong et al., 2017). Moreover, based on the fact that unlabeled or weakly labeled data are easier to obtain, another weakly supervised segmentation method has been proposed to make use of image-to-image translations to leverage unsegmented training data with and without cases of interest (Vorontsov et al., 2019). A simple and efficient way to randomly augment the training dataset, named InstraBoost, has been proposed using the existing human annotations through location probability map guided copying-and-pasting (Fang et al., 2019).

To boost network performance, a partially supervised multiorgan segmentation network has been implemented as a prior-aware neural network (PaNN) by explicitly incorporating anatomical priors on abdominal organ sizes; this network guides the training process with domain-specific knowledge (Zhou et al., 2019). In Roth et al. (2019), a distinct network architecture, along with a new training style, was carefully designed to assist the learning process, and thereby, the network was able to interpret errors made previously using automatically generated training labels. A few-shot segmentation network of foreground objects was demonstrated to give a support image and the ground-truth segmentation of the support image (Nguyen and Todorovic, 2019). The network’s performance is boosted by specifying its gradient for fine-tuning to new classes during the testing stage.

Our proposed method is different from previous work in that we first focused on the improvement of the quality of human annotations via well-designed fusion with network predictions, whereas the latter was exploited equivalently as “*a priori* knowledge.” Then, during boosting iterations, the sample weights of those hard-to-learn instances were sequentially updated until the desired network performance was obtained.

## Materials and Methods

In an endeavor to improve both the efficiency and accuracy of deep-learning-based methods for semantic segmentation of biomedical images while considering imperfections in human annotations, a boosting framework is proposed, as shown in Figure 1. We borrowed the word “boosting” from ensemble methods of machine learning to name the proposed framework since the base learner (i.e., the DCNN) within the framework was trained sequentially to pay more attention to instances with more segmentation errors.

The boosting framework consists of three major components, i.e., a multilabel DCNN (U-Net was used in this study but can be easily replaced by other networks) with the revised architecture to define a customized loss function (section “Multilabel Semantic Segmentation with U-Net With a Customized Loss Function”), a fusion module that aims to gradually fix the minor mistakes in human annotations based on network predictions without human intervention (section “Fusion Module”), and a boosting algorithm to sequentially update the sample weights during the network training iterations (section “Boosting Algorithm”). The performance of the boosting framework is assessed independently via a separate gold standard set, and thus, it can be trained and validated end-to-end until the desired performance is reached or the boosting iterations are exhausted.

### Multilabel Semantic Segmentation With U-Net With a Customized Loss Function

**FIGURE 2:**
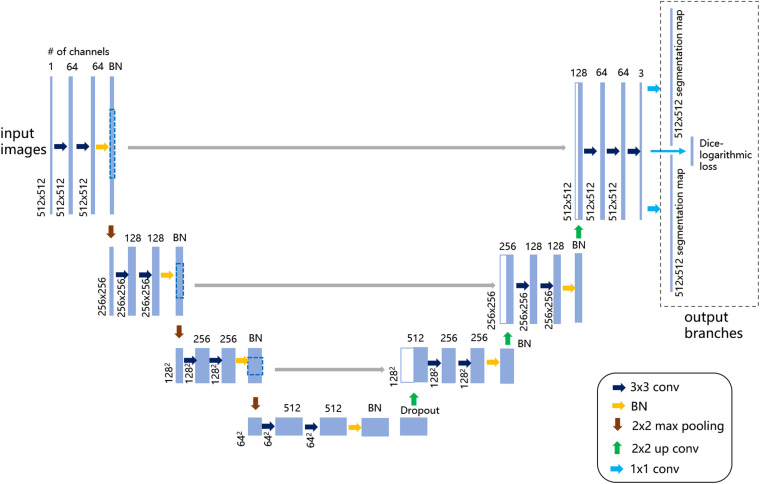
Illustration of the revised U-Net architecture for multilabel semantic segmentation. The figure demonstrates the architecture with the largest patch size of 512 × 512 voxels as input images on the left. Each light blue solid box corresponds to a multichannel feature map, as the channel number is denoted on top of the box and the image dimensionality is denoted on the left edge. The white boxes with light blue lines represent the concatenated copied feature maps from the contractive path. On the upper right, the dashed-line box represents the output branches consisting of two segmentation maps and one extra layer to compute the customized loss function. The colored arrows represent the different operations listed in the lower-right legend.

U-Net (Falk et al., 2019) was utilized as the base learner of the boosting framework. The network architecture was revised as in Figure 2 to produce multiple output layers, including two segmentation maps (e.g., somata and vessel structures in MOST) and an output layer for the computation of a customized Dice-logarithmic loss function. Moreover, after careful calibrations of the performance tuning, some extra layers, and hyperparameters used in the revised U-Net architecture were adjusted as described below.

**FIGURE 3:**
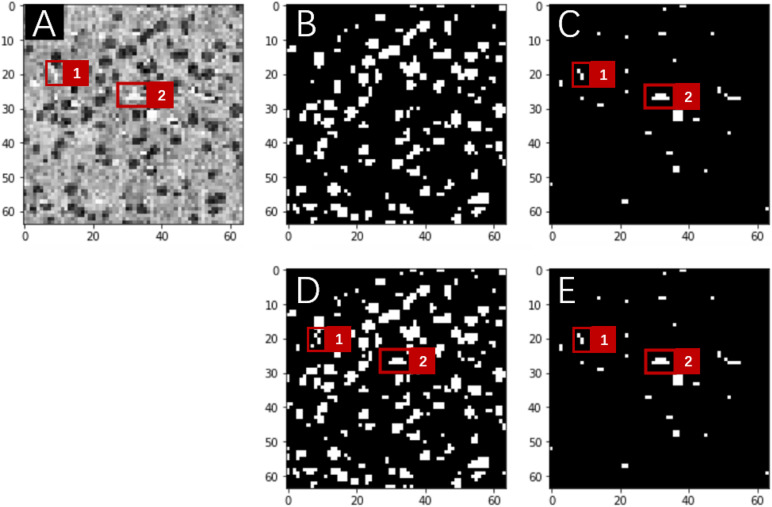
Network predictions vs. human annotations for a MOST 2D image (downsampling to 64 × 64 pixels) using the multilabel U-Net architecture with the standard cross-entropy loss function in a preliminary study. These preliminary studies were implemented with reduced image resolutions from the original MOST dataset for the purpose of fast explorations of parameter space. A frame from the raw image **(A)** contains somata (dark gray) and vessels (white), which are labeled in panels **(B,C)** and were predicted by the network in panels **(D,E)**, respectively. Red squares numbered 1 and 2 in panel **(A)** mark two vessel structures, which are annotated in panel **(C)** and were correctly predicted by the network in the “vessel” output layer [as in panel **(E)**] but mistakenly predicted in the “somata” output layer [as in panel **(D)**].

(1) Dropout and batch normalization (BN) layers
  To alleviate notorious overfitting problems in network training, our experiments based on the MOST dataset suggested that for a typical U-Net architecture, all BN layers were better placed symmetrically before pooling layers, and an extra dropout layer with a dropout rate of 0.5 was placed just before the upsampling layers.
(2) Customized Dice-logarithmic loss function
  The occurrence of somata in the MOST image stack was substantially more frequent than that of vessels, which suggests that for the purpose of network training, two classes of segmentation objects are not well balanced; i.e., it is likely that the network was trained with more information from somata than from vessels. Consequently, in multilabel segmentation tasks, the network output for the segmentation of one object sometimes contains the information from the other object even after hundreds of training epochs (Figure 3). This problem of ill-balanced classes across multiple segmentation objects is likely attributed to (1) predictions of both objects sharing the same network parameters except in the final output layer and (2) the standard cross-entropy loss function used for multiple segmentation outputs with ill-balanced classes probably being too slow to converge or even becoming trapped in some local minima. One possible solution is to implement a weighted loss function for all segmentation objects that regrettably introduces some extra hyperparameters (i.e., class weights) requiring additional laborious network fine-tuning. Another possible solution is focal loss (Lin et al., 2017), which, after some numerical experiments, did not substantially improve the segmentation performance in the proposed multilabel U-Net but introduced two more hyperparameters.

Instead, to mitigate the effects of ill-balanced classes in the training dataset without the introduction of extra hyperparameters, a novel loss function is proposed to maximize Dice coefficients (DCs) between network predictions and the corresponding annotations of multiple segmentation objects while simultaneously minimizing DCs across different objects. The latter was designed intentionally to prevent the network output for one object from containing information from other objects. Furthermore, the introduction of DCs into the loss function reconciled the metrics of network training and framework performance (Milletari et al., 2016).

We thus implemented a series of experiments on various forms of the DC-based loss function, among which a Dice-logarithmic loss has been shown to better help the convergence of the network training and defined as follows:

(1) $$\begin{array}{c} Loss_{total}=-\log\left[DC\left(Anno_{soma},Pred_{soma}\right)\right]\\ \;-\log\left[DC\left(Anno_{vessel},Pred_{vessel}\right)\right]\\ \;-\log\left[1-DC\left(Anno_{soma},Pred_{vessel}\right)\right]\\ \;-\log\left[1-DC\left(Anno_{vessel},Pred_{soma}\right)\right] \end{array}$$

where *Anno*_*soma*_ and *Anno*_*vessel*_ are the human-annotated labels for somata and vessels, respectively, in the MOST dataset, and *Pred*_*soma*_ and *Pred*_*vessel*_ are the corresponding network predictions. In addition, DC is defined as

(2) $$DC=\frac{2\left|Anno\cap Pred\right|}{\left|Anno\right|+\left|Pred\right|}.$$

The loss function defined in Equation (1) is calculated as one of the output layers in U-Net (Figure 1) and updated in real time during the network training stage.

### Fusion Module

Human annotations are not perfect for various reasons, e.g., too many similar objects with blurred boundaries and subtle differences between diminutive objects and the background. Thus, deep-learning networks trained from such datasets cannot be expected to fully segment the objects accurately. Furthermore, the assessment of network performance based on these “imperfect” datasets cannot be entirely reliable since the network might be misguided to learn something not even existing in the images.

Here, a fusion module is proposed to carefully and gradually “rectify” the errors in human annotations by reconciling the annotations with network predictions to better guide the training process of the deep-learning networks, which proves to be most likely data-driven. After the fusion, the “updated” human annotations were used as the new training set for the network. The general assumption behind the fusion module is that most of the human annotated errors for image segmentation tasks come with the missing or overlapped labels, and only a small part of the errors is attributed to fake labels, i.e., labels for an object that does not exist in the image.

**FIGURE 4:**
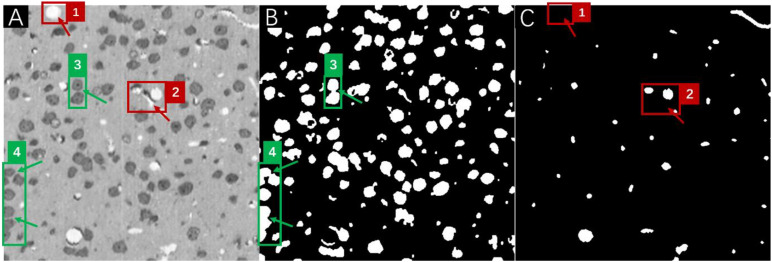
A frame of the MOST image stack **(A)** with the corresponding annotations for somata **(B)**, and vessels **(C)**. Red boxes numbered 1 and 2 in panel **(A)** mark vessel structures that were missed in human annotations (as pointed out by red arrows) in panel **(C)**, and green boxes numbered 3 and 4 in panel **(A)** mark soma structures that were annotated as overlapping labels (as pointed out by green arrows) in panel **(B)**.

Therefore, it is feasible to try to utilize the predictions of well-performing networks to locate and compensate for the missing/overlapped labels in the annotations under the conditions that (1) network performance should be good enough to provide meaningful corrections to the missing labels and (2) most of the human annotations are accurate and only a very small portion of the annotations contain errors such as missing and/or overlapped labels. The assumption could be readily verified in the MOST dataset, which after careful manual inspection indicated that nearly all errors in human annotations (about 95%) were attributed to missing and overlapping labels (Figure 4). The inspections also suggested that in principle, these missing/overlapped labels could be compensated by network predictions. However, due to the complexity of the determination of the “correct” object boundary from the overlapped labels between the annotations and the predictions, a simplified fusion solution to focus on missing labels is proposed in this work, and these missing labels likely were the major source of errors in the present multilabel semantic segmentation.

The fusion module was implemented for somata and vessel segmentation in the MOST dataset with three different fusion strategies; the three proposed strategies were as follows:

(a) the union of the predictions and annotations
(b) a combination of human annotations with missing labels located from the predictions
(c) a combination of network predictions with missing labels located from the annotations.

**FIGURE 5:**
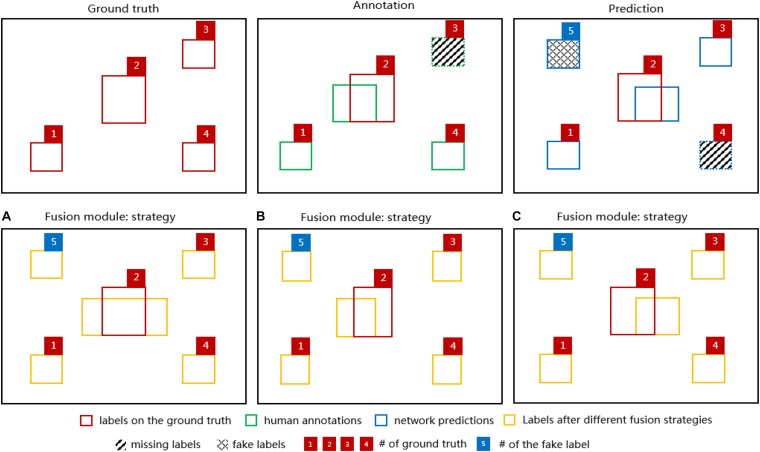
Diagrams of 4 segmentation labels of the ground truth (red boxes), the corresponding annotation (green boxes) and the network prediction (blue boxes) in the upper row. For demonstration purposes, there is a missing label in the annotation (green dashed-line box numbered 3) indicating human errors and a missing label (blue dashed-line box numbered 4) and a fake label (blue crossing-line box numbered 5) in the network prediction indicating network errors. In the lower row, the fusion module output (orange boxes) based on the diagrams above is shown for the fusion strategy **(A)**, **(B)**, and **(C)**. Note that one of the ground-truth labels (red box numbered as 2) has been added to each diagram to indicate the discrepancies between the ground truth and various labels.

The output of different fusion strategies (Figure 5) shows that considering that it is more likely for object labels to be missed in human annotations (e.g., green dashed-line box numbered as 3) and less likely for well-performed networks to predict fake labels (e.g., blue crossing-line box numbered as 5) completely, for MOST datasets, all three strategies were able to “recover” the missing labels either in the annotation or in the prediction after fusion. However, strategy (a) might be problematic and worthy of further investigation since it simply combines all possible errors from both the annotation and the prediction, and these errors would persist thereafter during network training. For strategies (b) and (c), the main difference is whether the prediction or the annotation would be used after fusion when overlapped labels occur.

The general algorithm in the fusion module is described in detail below.

First, the network was trained with the original MOST training set to achieve an overall DC performance of at least 0.90 for both somata and vessels (evaluated in a separate test set); otherwise, no fusion action occurs. Here, the performance threshold of 0.90 was chosen to ensure that network predictions would be good enough to guide the fusion algorithm in locating those missing labels.

Second, instances with DC less than 0.90 in the training set were selected to be fused with the corresponding network predictions based on the different strategies proposed above. More specifically, these selected human-annotated data were updated by comparison with the predictions to locate either those labels predicted by the network but missed in the annotations as in strategy (b) or those labels existing in the annotations but missed in the predictions as in strategy (c) using a modified “union-find” algorithm (Sedgewick and Wayne, 2011). Note that the missing labels were strictly defined without any overlapping with existing labels. Strategy (a) could be easily computed as a union operation between the annotation and the prediction.

Third, after fusion, the updated training set was utilized to train the network from scratch with the same training parameters. The network performance was then evaluated using a separate test set.

Our preliminary experiments of the proposed fusion module on the MOST dataset indicated that after 3000 epochs, the multilabel U-Net (as in section “Multilabel Semantic Segmentation with U-Net With a Customized Loss Function”) architecture was able to achieve an overall DC performance of about 0.95 in the training set and about 0.90 in the test set for both somata and vessels, and normally, less than 3% of training instances were fused with the corresponding network predictions.

### Boosting Algorithm

The fusion module ideally reduces the errors of human annotations in the training data, but its effectiveness heavily depends on the performance of the network, especially in hard instances, which would significantly impact the overall performance. After a few experiments in the MOST dataset, it was suggested that a one-time fusion between the annotations and predictions would likely not be enough for the network to achieve state-of-the-art (SOTA) performance. Multiple fusions might be more appropriate to gradually justify the network learning process. Thus, a boosting technique is proposed to allow the network to pay slightly more attention to the hard instances that the predecessor might underfit, causing the subsequent network to focus increasingly on the hard instances. The boosting algorithm works by following a similar fashion as the Adaboost method in conventional machine learning, with the main difference that the final ensembling stage in Adaboost is skipped in our framework because the instances in the training set are likely modified after each boost and fusion; thus, it might have been inappropriate to ask all the trained networks to vote the final results.

(1) As a result, the proposed boosting algorithm is implemented as followed: All the instances in the training data were initially assigned the same sample weight $w_{i}^{j}$ calculated as 1/*m*, where *i = 1…m* is the number of instances and *j* = 1 or 2 represents somata and vessels, respectively
(2) The network was trained with the weighted instances, and then, the DC was assessed for each instance for both somata and vessels. The weighted error rates *Err*_*j*_ were computed over the training set as
  (3) $$Err_{j}=\frac{\begin{array}{c} \sum_{\begin{array}{c} i=1\\ DC<\delta \end{array}}^{m}w_{i}^{j} \end{array}}{\sum_{i=1}^{m}w_{i}^{j}}$$
  where δ is the threshold value to determine which instances would be boosted. It was set as 0.97 for both somata and vessels.
(3) If *Err* was less than 0.5 but greater than 0, which means that the network performed well (i.e., a weak learner was at least more accurate than random guessing) in the training set, the sample weights could be updated as
  (4) $$w_{i}^{j}=\left\{ \begin{array}{ccc} w_{i}^{j} & if & DC\geq \delta \\ w_{i}^{j}*e^{0.5*\log\frac{1-Err_{j}}{Err_{j}}} & if & DC<\delta \end{array}\right.$$
  and then were normalized (i.e., divided by the sum of all weights). Otherwise, no sample weights were updated.
(4) The network was again trained from scratch with the instances of updated weights, and the whole process was repeated until the desired number of boosting iterations was reached or the goal of the network performance was achieved.

Since the network performance was normally evaluated by the test set during each boosting iteration to determine whether the network was properly trained, i.e., neither overfit nor underfit, another independent set was required to evaluate the efficiency of the boosting algorithm; i.e., to observe whether after boosting, the performance was de facto “boosted”. For this reason, an extra gold standard data set consisting of images was carefully inspected by a group of human experts to ensure that the annotations were as good as possible was introduced as an independent set (Figure 1). The network performance of multilabel semantic segmentation is thus reported based on both the test set and gold standard set after each boosting iteration.

To make predictions, the boosting algorithm simply employed the latest network after boosting iterations or the best-performing network evaluated on the gold standard set.

## Experiments and Results

The MOST system performs both thin slicing and imaging while recording the image coordinates for automatic alignment. By taking advantage of the modified Nissl staining method, the MOST system was able to provide a high-resolution data set of vascular and cellular structures of the entire mouse brain with a voxel size of 0.35 μm × 0.35 μm × 1 μm, interpolated to isotropic 0.35 μm and saved at a depth of 8 bits. The gray intensity of the voxel codes the cellular and vascular information of the brain (Wu et al., 2014). A representative MOST image stack of 512 × 512 × 1,000 voxels (i.e., 179 μm × 179 μm × 350 μm), along with the human annotations for both somata and vessels within the stack, were fed into the proposed boosting framework in this study. The annotations contained some errors mainly due to missing labels and inaccurate boundaries of somata and vessels. To independently assess the performance of the entire framework, a separate image dataset consisting of 10 2D images of MOST with labels that were carefully inspected and improved one-by-one by three human experts was utilized as the gold standard set. This small set of gold standards was never used during network training and validation. All the experiments were run within a Docker image configured with Ubuntu 16.04 LTS, Python 3.6 and Keras 2.2 with TensorFlow 1.14 as the backend on a Linux server equipped with 2 Nvidia 1080TI GPUs and 96 GB memory.

### Data Preprocessing

The MOST image stack, along with the 10 gold standard sets, was first processed via a technique named histogram equalization to enhance the contrast and was then normalized by simply dividing all pixel values by 255. The image stack along with the corresponding annotations for somata and vessels was randomly split into training, validation, and test sets, which consisted of 800, 100, and 100 images with the same voxel size of 512 × 512, respectively. Then, various data augmentation techniques (e.g., rotation, shifting, zoom-in/out, flipping, etc.) were applied to the original training set (i.e., 800 images) to generate the final set of 2,400 images ready for network training. No real-time data augmentation was applied thereafter during the boosting iterations.

### Network Training Strategy

The training and validation sets of the MOST image stack were fed into the U-Net (as described in section “Multilabel Semantic Segmentation With U-Net with a Customized Loss Function”) and trained for 3,000 epochs using the Adam optimizer with a constant learning rate of 5×10^−5^. The epoch number of 3,000 was carefully selected since a few numerical experiments indicated that the U-Net performance evaluated in the training and validation sets was nearly stable after 3,000 epochs. During the training, each instance was initially assigned a sample weight of ½,400. The best trained network was selected based on the performance in the validation set by observing the customized Dice-logarithmic loss after each epoch and then was evaluated in the test set of 100 images and the gold standard set of 10 images.

### Fusion and Boosting

Depending on the performance of the best trained network in the test set, the training instances that were selected according to the threshold values described in section “Fusion Module” were fused with the corresponding predictions, resulting in a new training set. Then, the sample weights for the new training set were updated according to the boosting algorithm described in section “Boosting Algorithm.”

This new training set and the updated sample weights were fed into the U-Net architecture to train from scratch for 3,000 epochs. The best-trained network was again utilized for the fusion and boosting iteration. This whole process was repeated 10 times within the proposed boosting framework.

### Results Analysis

**FIGURE 6:**
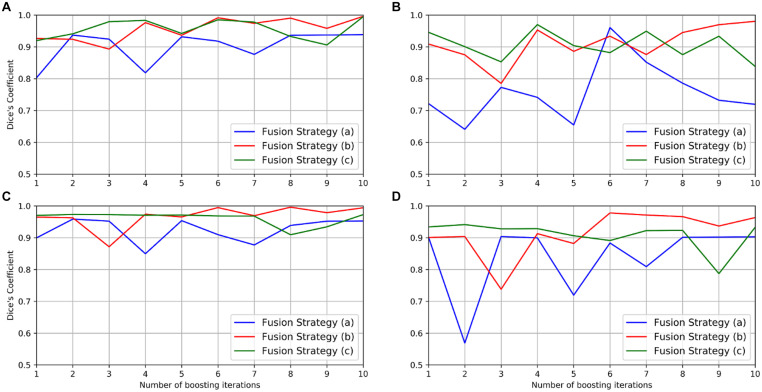
Performance of the boosting framework during 10 iterations: mean DC for somata **(A)** and vessels **(B)** in the gold standard set. The results from three different fusion strategies are indicated by blue, red, and green solid lines. The performance on the test set is also shown for somata **(C)** and vessels **(D)**.

The performance of the boosting framework assessed via mean DC in the gold standard and the test set with different fusion strategies is shown in Figure 6 and summarized in Table 1. The fusion strategy (a) performed poorly, especially in the segmentation of vessels in the gold standard set (mean DC as 0.719). The fusion strategy (c) performed relatively well for the segmentation of somata in both the gold standard (mean DC as 0.994) and the test set (mean DC as 0.972) but worse than the fusion strategy (b) for the segmentation of vessels (mean DC as 0.838 vs. 0.971, and 0.933 vs. 0.963, respectively).

**TABLE 1:** Network performance (mean DC) and performance boosting trend (linear coef.) of the proposed framework.

Models		Object	Gold Standard DC/Linear Coef	Test Set DC/Linear Coef.
DDeep3M		Soma	0.988	0.968
		Vessel	0.937	0.923
U-Net without fusion/boosting		Soma	0.956	0.964
		Vessel	0.825	0.912
Boosting framework	Strategy (a)	Soma	0.938/0.0087	0.953/0.0024
		Vessel	0.719/0.0080	0.903/0.0013
	Strategy (b)	Soma	**0.996/0.0085**	**0.994/0.0036**
		Vessel	**0.971/0.0012**	**0.963/0.0082**
	Strategy (c)	Soma	0.994/0.0013	0.972/-0.0035
		Vessel	0.838/-0.0043	0.933/-0.0069

*The performance boosting trend is indicated by linear coefficients fitted by the linear regression model for different fusion strategies. Positive values in the linear coefficient indicate a general upward trend, and negative values indicate a downward trend. For comparison, the performance of DDeep3M and multilabel U-Net (trained for 30,000 epochs without fusion and boosting iterations) is also shown. The bold values mean that within the same column, they represent the best results in various experiments.*

To observe whether the boosting framework indeed improves network performance, a linear regression model was then employed to analyze the trend of the network performance by fitting the mean DC in the gold standard and the test set, respectively, during boosting iterations. The result indicated that both fusion strategies (a) and (b) “boosted” the network performance with more iterations since the linear coefficients were positive, whereas no such consistent performance improvement was observed in the fusion strategy (c) (Table 1).

Therefore, the fusion strategy (b), i.e., a combination of human annotations with missing labels located from the predictions, is likely the better option in the MOST dataset. More specifically, the mean DC in the fusion strategy (b) for the gold standard set is “boosted” from 0.927/0.886 (without fusion and boosting) to 0.996/0.971 for somata and vessels, respectively. Performance boosting was also observed in the test set, i.e., from 0.965/0.903 to 0.994/0.963 for somata and vessels, respectively. To further verify the effectiveness of the fusion and boosting algorithm, the multilabel U-Net architecture used in the framework was trained independently for 30,000 epochs (set to the total epoch number after 10 boosting iterations) using the same learning rate (i.e., 5×10^−5^) but without fusion and boosting. The result (as shown in the last column of Table 1) indicates that the proposed framework improves network performance in the MOST dataset, whereas performance is improved substantially more for vessels and less for somata.

Compared with the previous network (DDeep3M; Wu et al., 2020), the proposed framework (after 10 boosting iterations) achieved slightly better performance in the same dataset (0.996 vs. 0.988 for somata and 0.971 vs. 0.967 for vessels) and outperformed significantly in terms of training duration (6 h vs. 36 h) and prediction speed (1 s vs. 24 s on a 1,024 × 1,024 MOST 2D image). Additionally, in a study of deep-learning-based analysis of whole mouse brain vasculature at the micrometer scale (Todorov et al., 2020), a transfer-learning approach was employed to increase the performance of the network, for which similar performance on the segmentation of vessels was reported with an accuracy of 0.94 ± 0.01 for VesSAP CNN, 0.95 ± 0.01 for 3D U-Net, and 0.95 ± 0.02 for V-Net (Milletari et al., 2016).

**FIGURE 7:**
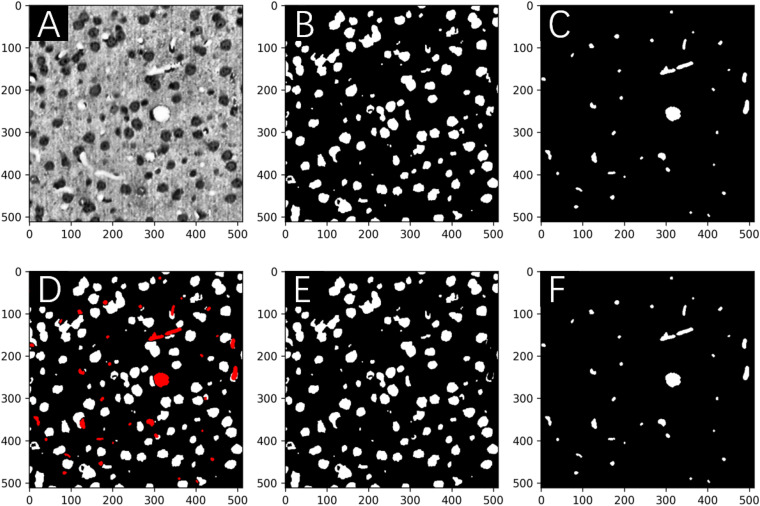
Network predictions *vs*. human annotations for a MOST 2D image in the gold standard set in the boosting framework after 10 iterations. Image **(A)** is labeled for somata **(B)** and vessels **(C)** and then predicted by the network in panels **(E,F)**, respectively. The predictions are merged into one image **(D)**, where the vessels are shown in red. The DC for the segmentation of somata and vessels was 0.994 and 0.963, respectively.

**FIGURE 8:**
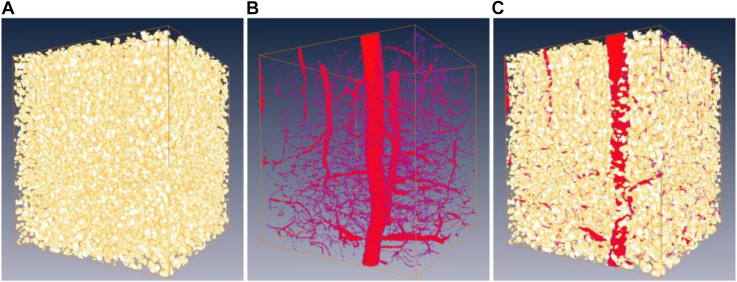
Predictions on a 3D MOST image stack by the boosted network. Panels **(A,B)** are the predictions of somata (shown as gold standard) and vessels (shown as red). **(C)** 3D reconstruction of somata and vessels by merging **(A,B)**.

Overall, the proposed framework improves the network performance by *about* 3–10% for both somata and vessels, even considering that minor errors occur in human annotations of the MOST dataset. As an example, a representative frame from the gold standard set is shown in Figure 7 with the corresponding predictions from the network after 10 iterations. Finally, the boosted network was utilized for the semantic segmentation of somata and vessels in an independent MOST image stack of 1,024 × 1,024 × 1,024 voxels (i.e., 358 μm × 358 μm × 358 μm), and the results could be merged into one single block (Figure 8) for 3D digital reconstruction of the mouse brain, which could be used to explore the neurovascular network.

## Discussion and Conclusion

In this work, we have proposed a boosting framework, combining a U-Net architecture with a customized loss function, a fusion module, and a boosting algorithm, to systematically improve the quality of the human annotations that eventually resulted in a performance boost in the multilabel segmentation task using DCNNs. The framework was assessed using a MOST image stack for a segmentation task of somata and vessels. Evaluation with an independent gold standard set of 10 images revealed that the framework significantly increased the segmentation performance of U-Net from 0.927 to 0.996 for somata and 0.886 to 0.971 for vessels. An overall performance improvement of 7% was achieved after 10 boosting iterations in this semantic segmentation task for the MOST image stack. In comparison with the existing SOTA segmentation solutions for MOST image stacks, which report about 0.986/0.967 in DC (Wu et al., 2020), the proposed framework achieves slightly better performance with less time and demonstrates its power even with poor-quality data.

Some limitations exist in our work. The number of gold standard sets was probably not enough to thoroughly assess the performance of the boosting framework, and we did not perform sensitivity analyses with respect to the variations of gold standard sets. 2D U-Net was used but was applied to essentially a three-dimensional segmentation task based on MOST image stacks. We expect that the DCNN in the framework can be easily replaced by other networks, such as 3D U-Net. The fusion module mainly focuses on the recovery of missing labels but does not provide a comprehensive algorithm to reconcile overlapped labels among the ground truth, the annotation and the prediction. The number of boosting iterations (i.e., 10) was manually selected, which should be adjusted in real time based on the performance goal in future work.

Our work substantially lowers the requirement of time-consuming high-quality human annotations, which normally are the key to the success of DCNNs in segmentation tasks; thus, this work would greatly help researchers who are eager to utilize deep learning technology but are limited by the amount of high-quality data. In principle, the boosting framework may be scaled up to the whole-brain level for both somata and vessels (Todorov et al., 2020). Precise segmentation is directly helpful for quantitative analyses of neurovascular networks (Wu et al., 2014). This proposed technique of artificial intelligence could advance basic neuroscience research.

## Data Availability Statement

The proposed framework, along with MOST sample data, is open-sourced and publicly available from GitHub under the Apache License 2.0. Detailed implementation of the framework, including the U-Net, the fusion module, the boosting algorithm, and the network training and validation process, is described in a GitHub repository as https://github.com/cakuba/Boosting_multi-label_semantic_segmentation.

## Ethics Statement

The animal study was reviewed and approved by Institutional Animal Ethics Committee of Huazhong University of Science and Technology.

## Author Contributions

XW: conceptualization, methodology, and project administration. SC and JH: funding acquisition and supervision. YT, GH, DL, MF, and SY: coding and experiment. HG: funding acquisition and writing—review and editing. RX: data curation and writing—review and editing. All authors contributed to the article and approved the submitted version.

## Conflict of Interest

The authors declare that the research was conducted in the absence of any commercial or financial relationships that could be construed as a potential conflict of interest.
